# A pediatric death audit in a large referral hospital in Malawi

**DOI:** 10.1186/s12887-018-1051-9

**Published:** 2018-02-21

**Authors:** Elizabeth Fitzgerald, Rachel Mlotha-Mitole, Emily J. Ciccone, Alyssa E. Tilly, Jennie M. Montijo, Hans-Joerg Lang, Michelle Eckerle

**Affiliations:** 1 Assistant Professor of Pediatrics, University of North Carolina at Chapel Hill, Chapel Hill, USA; 2 University of Malawi College of Medicine, Blantyre, Malawi; 3 University of North Carolina at Chapel Hill, Chapel Hill, USA; 4 Hawaii Permanente Medical Group, Maui, Hawaii USA; 5 Médecins sans Frontières – Belgium, Paediatric Referent, Brussels, Belgium; 6 Assistant Professor of Pediatrics, Cincinnati Children’s Hospital Medical Center, Cincinnati, Ohio USA

**Keywords:** Pediatric emergency medicine, Global Health, Death audits

## Abstract

**Background:**

Death audits have been used to describe pediatric mortality in under-resourced settings, where record keeping is often a challenge. This information provides the cornerstone for the foundation of quality improvement initiatives. Malawi, located in sub-Saharan Africa, currently has an Under-5 mortality rate of 64/1000. Kamuzu Central Hospital, in the capital city Lilongwe, is a busy government referral hospital, which admits up to 3000 children per month. A study published in 2013 reported mortality rates as high as 9%. This is the first known audit of pediatric death files conducted at this hospital.

**Methods:**

A retrospective chart review on all pediatric deaths that occurred at Kamuzu Central Hospital (excluding deaths in the neonatal nursery) during a 13-month period was done using a standardized death audit form. A descriptive analysis was completed, including patient demographics, HIV and nutritional status, and cause of death. Modifiable factors were identified that may have contributed to mortality, including a lack of vital sign collection, poor documentation, and delays in the procurement or results of tests, studies, and specialist review.

**Results:**

Seven hundred forty three total pediatric deaths were recorded and 700 deceased patient files were reviewed. The mortality rate by month ranged from a low of 2.2% to a high of 4.4%. Forty-four percent of deaths occurred within the first 24 h of admission, and 59% occurred within the first 48 h. The most common causes of death were malaria, malnutrition, HIV-related illnesses, and sepsis.

**Conclusions:**

The mortality rate for this pediatric referral center has dramatically decreased in the 6 years since the last published mortality data, but remains high. Areas identified for continued development include improved record keeping, improved patient assessment and monitoring, and more timely and reliable provision of testing and treatment. This study demonstrates that in low-resource settings, where reliable record keeping is often difficult, death audits are useful tools to describe the sickest patient population and determine factors possibly contributing to mortality that may be amenable to quality improvement interventions.

## Background

The path to pediatric mortality in the developing world is complex, with myriad modifiable factors that can serve as public health intervention points. Programs directed at reducing child mortality have traditionally focused on the areas of prevention and improving access to care. More recently, the impact that acute and inpatient hospital care has on child mortality has been recognized, and opportunities for improvement have been described [1–5]. To successfully implement care improvement plans, existing systems must be objectively assessed and components amenable to intervention identified. However, accurate record keeping is often a challenge in low-resource settings. The paucity of reliable information about in-hospital care leads to further challenges in evaluating barriers to treatment, and in creating and assessing quality improvement interventions. The lack of valid vital statistics in the developing world often leaves many deaths unaccounted for, and the lack of accurate information regarding patient’s cause of death complicates the ability to plan for, fund, and assess interventions [6, 7]. Audits of deceased pediatric patient files have been shown to be a useful tool in creating sustainable hospital Quality Improvement initiatives in low-resource settings [8–10].

Malawi is a resource-poor, malaria-endemic country in sub-Saharan Africa, consistently ranked among the least developed in the world. Approximately 62% of its 16.7 million inhabitants live below the international poverty line of $1.25 USD (US Dollar) per day. Despite its challenges, great strides have been made in improving the health of children in Malawi in the last few decades. Malawi has reached its fourth Millennium Development Goal, a two-thirds reduction in childhood mortality, before the target date of 2015 [11]. The World Health Organization (WHO) reports that Under-five mortality was 71/1000 in 2013, compared to 245/1000 in 1990. However, although Malawian pediatric patients are accessing clinical care at an increasing rate, the clinical standards set forth by the WHO for some common illnesses are being inconsistently adhered to in hospitals nationwide [11].

Kamuzu Central Hospital (KCH) is a tertiary referral hospital in Lilongwe, Malawi, which serves the central region of the country and a population of approximately 5 million people. The pediatric ward of the hospital admits between 40 and 120 patients per day, with significant seasonal variation related to malaria burden and food insecurity. Medical record keeping has traditionally been inconsistent; charts are hand-written when paper is available and held by the pediatric patient’s caregiver. There is no electronic medical record. Deaths are recorded in a ledger by an administrative layperson, with a presumed cause of death as diagnosed by the evaluating clinician. To date, no comprehensive effort has been made at investigating modifiable factors that may contribute to pediatric mortality at KCH. We report the results of the first known audit of pediatric death files conducted at this hospital. In the absence of reliable record-keeping, the objective of this study was to describe the patients who died in the hospital, evaluate the emergency and inpatient care provided to them, and identify gaps in their care that may have contributed to their mortality.

## Methods

A retrospective chart review was conducted by a team of two pediatricians and two University of North Carolina (UNC) Medicine-Pediatric residents on behalf of the Pediatric Department at KCH. Each member of the team was responsible for the review of a portion of the death files, and the entry of the collected data into an Excel spreadsheet. Hospital charts for all pediatric deaths that occurred at Kamuzu Central Hospital during a 13-month period were reviewed using a standardized death audit form, created and approved by the KCH medical staff for data collection. The data was de-identified and recorded in an electronic database. Variables extracted are listed in Table 1. Data collected included patient date of birth or approximate age (if the patient’s date of birth was unknown), gender, address, date and time of admission and death, HIV and immunization status, nutritional state, and presumed cause of death. Patient weights were frequently estimated by clinicians, and it was uncommon for a formal nutritional assessment to have been completed. Therefore, in order to avoid over-estimating the impact of malnutrition, nutritional state was assumed to be normal unless a formal nutritional assessment indicating malnutrition had been completed, or a provider had clinically assessed the patient to be malnourished. Cause of death was extrapolated based on recorded information, but was often difficult to definitively diagnose given the limited diagnostic tools. A combination of recorded history, including the diagnosis recorded by the clinician who declared the patient dead, physical exam findings, and test results were used to infer the cause of death. If a patient had an underlying disease process such as HIV or malnutrition and a secondary illness such as malaria, the cause of death was considered to be the primary disease process. Additionally, we reviewed the initial patient assessment and noted whether any vital signs were recorded at first encounter. The pediatric ward admission sheet contains an assessment tool meant to approximate an Emergency Triage and Assessment Tool (ETAT) evaluation and determine patient acuity. Percent completion of this form was reviewed. Further data was collected about whether any vital signs were recorded during the first twenty-four hours of hospitalization, and whether a complete set of vital signs (temperature, heart rate, respiratory rate, oxygen saturation) was obtained at least once each day during the hospitalization. A complete review of each chart was then conducted, with a focus on identifying delays in the assessment or management of patients. We specifically noted delays in the procurement of radiology studies, specialist consultation, nutritional assessment and treatment, initiation of TB treatment, and HIV testing. A delay was defined as greater than 24 h between the ordering and completion of a diagnostic test, evaluation, or treatment. Ethical approval was not required, as this was considered a KCH Pediatric department audit.

**Table 1:** Patient Characteristics (*N* = 700)

	Number (%)
Age	
Median	24 months
Range	1 day to 16.5 years
Gender	
Male	353 (50.4)
Female	353 (50.4)
Unknown	14 (2)
Nutritional status	
Unknown (not documented)	535 (76.4)
Newborn	24 (3.4)
Malnourished	141 (20)
Marasmus	65 (9.3)
Kwashiorkor	36 (5.1)
Marasmic Kwashiorkor	31 (4.4)
Unknown type	9 (1.3)
HIV status	
Non-reactive	222 (31.6)
Reactive	40 (5.7)
Unknown	413
Deceased on arrival	86 (12.2)
Died before testing could be completed	204 (29)
Untested	126 (18)
Presumed Severe HIV disease	24 (3.4)
Immunization status	
Unknown (undocumented)	495 (70.9)
Vaccines up to date	82 (11.7)
Vaccines deficient	4 (0.6)
Not applicable (neonate)	33 (4.7)
Deceased on arrival	86 (12.2)
Referral History	
Referred from outside health facility	464 (66.3)
Referral note in chart	100 (21.4)
No referral note	333 (71.3)
Unknown	31 (6.8)
Self-referred	157 (22.4)
Unknown	79 (11.3)

## Results

**Fig. 1:**
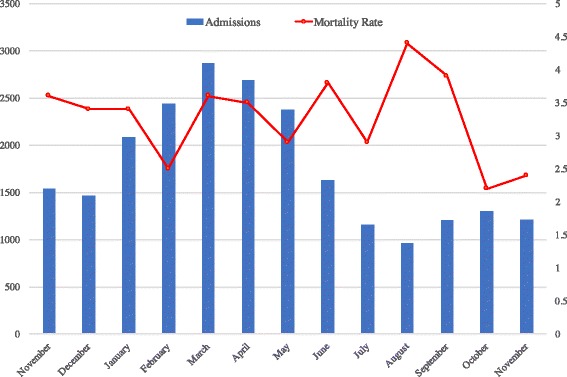
Admissions and deaths in the Pediatric department at KCH November 2014 to November 2015

A total of 743 total pediatric deaths were recorded in the hospital death registry during the audit period, and 700 deceased patient files were reviewed. Forty-three charts (5.8%) were missing and unable to be reviewed. Total inpatient admissions to the pediatric ward ranged from 956 to 2866 per month (Fig. 1). The monthly mortality rate ranged from a low of 2.2% in October 2015 to a high of 4.4% in August 2015. The mean was 3.3%. Patient ages at admission ranged from 1 day to 16.5 years, with a median age of 24 months. Eighty-six patients were brought to the hospital already deceased. Thirty-seven percent of deaths occurred within the first 24 h of admission, and 53% occurred within the first 48 h. The nutritional status of the majority of the patients was not noted in the chart, but 19% were either reported by the clinician or assessed by the Nutritional Rehabilitation Unit to have some form of malnutrition. Kwashiorkor was the most prevalent form. HIV status was determined in 38% of patients, with 5.7% positive and a further 4% with presumed severe HIV disease based on their mother’s HIV status and the clinical criteria determined by the WHO Staging guidelines. No vital signs were done at initial presentation in 44% of patients, and 49% did not have any recorded vital signs within the first twenty-four hours of hospitalization (Table 2). Documentation deficiencies were noted in 58% of the charts reviewed, with missing admission sheets being the most common deficiency (16%). Thirteen percent of patients were missing a note, and presumably a review, from a clinician on at least one day of hospitalization. There were one hundred and two cases where at least one radiology study was ordered and not completed within 24 h, most frequently chest X-rays and ultrasounds of the heart or abdomen. Of the 392 patients alive at 24 h after admission, 56 (14.3%) had an HIV test ordered but not done within 24 h. There was a delay of greater than 24 h in obtaining a Nutritional Rehabilitation Unit (NRU) evaluation or consultant review in 13% and 11% of cases, respectively. The most frequent consultant delay was in surgical review. The most common cause of death was malaria, with malnutrition, HIV, and sepsis being the next most common causes of death (Table 3).

**Table 2:** Characteristics of care provided

	Number (%)
Vital signs obtained at admission	
At least one vital sign obtained	303/614 (49.3)
Temperature	212 (34.5)
Heart rate	203 (33.0)
Respiratory rate	101 (16.4)
Oxygen saturation	219 (35.6)
Blood pressure	25 (4.1)
No vital signs obtained	311 (50.1)
Use of the initial assessment tool	
Some use	439/614 (71.2)
Not used	175 (28.5)
Vital signs obtained within the first 24 h of admission	
At least one vital sign obtained	343/614 (55.9)
Temperature	249 (40.1)
Heart rate	262 (42.7)
Respiratory rate	98 (16)
Oxygen saturation	304 (49.5)
Blood pressure	23 (3.7)
No vital signs obtained	271 (44.1)
Complete set of vital signs obtained at least once each 24 h	
At least one complete set per day	154/614 (25)
At least one day without a complete set of vital signs	462 (75.2)
Delays in assessment/care	
HIV testing	56/392 (14.3)
NRU assessment	51/392 (13)
TB treatment	26/392 (6.6)
Specialist consultation	43/392 (11)
Radiology studies	102/700 (14.6)

**Table 3:** Presumed Cause of Death

	Number (%)
Malaria	183 (26.1)
Malnutrition	95 (13.6)
HIV-related illness	69 (9.9)
Sepsis	62 (8.9)
Unknown	39 (5.6)
Respiratory disease	35 (5.0)
Meningitis/encephalitis	34 (4.9)
Perinatal death	31 (4.4)
Gastroenteritis	27 (3.9)
Heart disease	25 (3.6)
Surgical complication/missed surgery	22 (3.1)
Cancer	19 (2.7)
Anemia/blood disorder	18 (2.6)
Renal failure	13 (1.9)
Seizures	10 (1.4)
Trauma/burns	9 (1.3)
Tetanus/poisoning	7 (1.0)
Liver failure	2 (0.3)

## Discussion

Worldwide, there are large numbers of pediatric patients presenting to hospitals for emergency care, most of whom are previously healthy [12, 13]. Improvement in the mortality of these patients contributes to life-years saved and productivity. Yet it is well-recognized that there is a lack of basic data in emergency settings in low- and middle-income countries (LMCI’s), and that this has made the assessment of systems and identification of priorities challenging [12–14]. As was noted by the World Health Organization in 2005, “epidemiology, demography and biostatistics are the key disciplines of public health,” and lack of data, especially in the inpatient setting, has hampered progress [7]. In 2010, eight agencies in Global Health put out a call to action on health data, specifically including health-facility reporting, to inform decision-making and assess interventions [6]. Use of death audits is one approach that has been used to evaluate hospital care when poor record-keeping, high volume, and inadequate staffing make assessment difficult [9, 10, 15]. Kamuzu Central Hospital is no exception to the challenges of monitoring and evaluation of its pediatric systems. This retrospective review of the files of deceased pediatric patients at KCH was undertaken in an effort to describe the care provided to a cohort of patients who presented to the pediatric department emergently, and identify gaps in their care.

The results of this retrospective 13-month review of pediatric death audits reveal that mortality at KCH has dramatically improved since the last published review in 2012, when it was as high as 9.3% [16], however it still remains significant, with an average monthly mortality of 3.3%. Without more information about the number of admissions and the care provided during the 2012 study, it is difficult to directly compare these statistics. A number of interventions have occurred in the pediatric department since that publication, including improved consultant-level coverage and a restructuring of the triage and emergency areas, which may have contributed to this substantial decrease in inpatient mortality. Further efforts should be made to investigate and describe the changes made, and to determine the impact that these efforts may have had on mortality.

The mortality rate in our study was somewhat variable, with a monthly range from 2.2% to 4.4%, and surprisingly the month with the highest rate of deaths was not the one with the highest number of admissions. This mortality rate is similar to one seen in a 2015 systematic review of emergency care in LMCI’s that showed the median pediatric mortality rate among fourteen sub-Saharan African hospitals was 5.1% [12]. The majority of deaths in our review occurred in patients younger than 24 months, and most occurred within the first 48 h of admission. Multiple studies of inpatient mortality in pediatric public hospitals in Africa have demonstrated similarly that the majority of deaths occur within the first hours of admission [17–19]. It is unusual that there were not more neonatal deaths reported, however this is likely due to the infrastructure of the pediatric department at KCH. Most neonatal deaths occur in the newborn ward or the infant nursery, and so information about their care is not accurately reflected in this review. Inclusion of this data would certainly both increase the overall mortality rate and change the age distribution of deceased patients.

This investigation reveals multiple modifiable factors that, if addressed, may reduce pediatric inpatient mortality at Kamuzu Central Hospital. Vital sign collection was often inadequate, a finding demonstrated in previous descriptions of pediatric acute care facilities in LMIC’s. A 2001 review of 21 such hospitals demonstrated that 30% of inpatients were not monitored or reassessed [13]. It is difficult to prove definitively that inadequate monitoring was associated with mortality without reviewing the charts of age- and disease-matched controls, an effort that was beyond the scope of our review. However, earlier and more frequent patient assessment has been demonstrated to improve outcomes [20].

Multiple studies have shown the benefit of early recognition and treatment of critically ill children through use of Emergency Triage Assessment and Treatment [16, 19, 21, 22]. Our review showed that, despite inclusion of an initial assessment tool on the admission form (meant to approximate an ETAT assessment), documentation of a complete initial evaluation was inconsistent. Previous research has shown that interventions as simple as providing a pre-printed standardized admission form improves data collection [14], and it was rare that any of the 115 charts with missing admission forms contained any initial vital signs or ETAT assessment. It is possible that assessments of our patients were completed but not documented.

Nutritional review of our patients, including an accurate weight and Mean Upper Arm Circumference (MUAC), was rarely completed, despite the fact that nutritional status is a key factor associated with morbidity and mortality in the developing world [23, 24]. When malnutrition was identified by a clinician, there were often delays in initiating nutritional therapy. Our decision to exclude patients who did not have a formal nutritional assessment completed or who were not noted by the clinician to be malnourished likely underestimated the degree to which poor baseline nutritional status contributed to mortality. Similarly, HIV status was not determined in a significant number of children, although the evidence supporting the routine testing of inpatients is clear [25, 26]. It is possible that patients who were not tested were HIV reactive, and that our assessment of the contribution of HIV to the mortality rates of our patient population was underestimated.

Referral notes from providers outside the hospital were often missing from the chart, and documentation deficiencies were frequent. There were multiple instances of delays in the procurement of radiology studies, initiation of TB treatment, initiation of NRU assessment and treatment, and consultant review. Comparing the outcomes of a matched cohort of patients for whom timely care was provided would strengthen the implication that these delays contributed to mortality, but that effort was beyond the scope of our study due to the high number of patient charts that would need to be reviewed.

Determining the actual cause of death in a setting where clinicians are unable to perform diagnostic tests is inherently difficult. With such limited access to radiologic or laboratory investigations, cause of death is often based on clinical judgement, and may be inaccurate [27]. The diagnosis of sepsis is often made in patients with undifferentiated shock when more accurate diagnoses cannot be made, and is likely over-estimated in our review. For similar reasons, the contribution of malaria to the mortality rates is likely overestimated. The diagnosis of malaria at KCH is typically made based on positive Malaria Rapid Diagnostic Testing (MRDT), as peripheral smears are more difficult to obtain. These positive MRDTs could simply reflect a recent infection, and do not confirm that malaria was the cause of death [28, 29]. Future studies that more specifically diagnose malaria based on plasma PfHRP2 concentration, or that include confirmatory evaluation of parasite density on peripheral blood smears, would certainly enhance understanding of the extent to which malaria contributes to mortality at this hospital.

Finally, our decision to base the cause of death on known primary disease processes such as HIV or malnutrition, when there were often other etiologies that contributed to their deaths, may overestimate their contribution to patient mortality rates.

Although this audit clearly demonstrates several opportunities for improvement, it is also likely that the data underestimates the need, while simultaneously serving as a reminder of the “real-world” conditions in under-resourced settings. The poor quality of patient charts makes evaluation difficult, as data are often missing or illegible [6]. Furthermore, this data reflects delays in completion of requested interventions, but we did not measure delays in ordering studies, treatments, or specialist care. Our review does not include delays or deficiencies in medication administration or laboratory testing, which are essential components of treatment whose measurement was beyond the scope of this study, but which have been demonstrated to be deficient in similar setting [13, 30, 31]. Further chart reviews, including a comprehensive review of the care provided to and mortality rates in the newborn nursery, need to be done in order to render a more complete assessment of the quality of pediatric care and possible areas for intervention. This study should serve as the first step in what must be an ongoing process of hospital evaluation, so that modifiable barriers to standards of care can be continually identified, efforts at improvement can be rendered, and the impact of interventions can be demonstrated. As stated in the 2005 WHO Bulletin, “It is not because countries are poor that they cannot afford good health information; it is because they are poor that they cannot afford to be without it.” [7].

## Conclusion

The use of death audits to evaluate inpatient care in low and middle-income countries, where record keeping is often variable or inadequate, has proven to be beneficial. This study demonstrates improved mortality at a busy pediatric referral center in central Malawi, but also reveals gaps in care that may be contributing to the high percentage of deaths that occur in the first 48 h of admission. Identified gaps include poor documentation, inadequate patient assessment and monitoring, and delays in standard care.

## Abbreviations

ETAT: Emergency Triage and Assessment Tool
HIV: Human Immunodeficiency Virus
KCH: Kamuzu Central Hospital
MUAC: Mean Upper Arm Circumference
NRU: Nutritional Rehabilitation Unit
TB: Tuberculosis
UNC: University of North Carolina at Chapel Hill
USD: US Dollar
WHO: World Health Organization
